# Auranofin is a potent suppressor of osteosarcoma metastasis

**DOI:** 10.18632/oncotarget.5704

**Published:** 2015-11-09

**Authors:** Eleni Topkas, Na Cai, Andrew Cumming, Mehlika Hazar-Rethinam, Orla Margaret Gannon, Melinda Burgess, Nicholas Andrew Saunders, Liliana Endo-Munoz

**Affiliations:** ^1^ The University of Queensland Diamantina Institute, The University of Queensland, Translational Research Institute, Brisbane, Queensland, Australia

**Keywords:** auranofin, metastasis, thioredoxin reductase, osteosarcoma, oxidative stress

## Abstract

Osteosarcoma (OS) accounts for 56% of malignant bone cancers in children and adolescents. Patients with localized disease rarely develop metastasis; however, pulmonary metastasis occurs in approximately 50% of patients and leads to a 5-year survival rate of only 10–20%. Therefore, identifying the genes and pathways involved in metastasis, as new therapeutic targets, is crucial to improve long-term survival of OS patients. Novel markers that define metastatic OS were identified using comparative transcriptomic analyses of two highly metastatic (C1 and C6) and two poorly metastatic clonal variants (C4 and C5) isolated from the metastatic OS cell line, KHOS. Using this approach, we determined that the metastatic phenotype correlated with overexpression of thioredoxin reductase 2 (*TXNRD2*) or vascular endothelial growth factor (*VEGF*). Validation in patient biopsies confirmed *TXNRD2* and *VEGF* targets were highly expressed in 29–42% of metastatic OS patient biopsies, with no detectable expression in non-malignant bone or samples from OS patients with localised disease. Auranofin (AF) was used to selectively target and inhibit thioredoxin reductase (TrxR). At low doses, AF was able to inhibit TrxR activity without a significant effect on cell viability whereas at higher doses, AF could induce ROS-dependent apoptosis. AF treatment, *in vivo*, significantly reduced the development of pulmonary metastasis and we provide evidence that this effect may be due to an AF-dependent increase in cellular ROS. Thus, TXNRD2 may represent a novel druggable target that could be deployed to reduce the development of fatal pulmonary metastases in patients with OS.

## INTRODUCTION

Osteosarcoma (OS) accounts for 56% of malignant bone cancers and 6% of all cancer cases in children and adolescents [1]. OS patients with localised disease have an expected 5-year survival rate of approximately 70% [2]. However, pulmonary metastasis occurs in approximately 50% of patients and leads to a 5-year survival rate of less than 20% [2]. OS patients with metastatic disease do not respond to currently available treatments, thus presenting a significant challenge to the clinical management of the disease [2]. Therefore, the identification of genes and pathways that drive the metastatic behaviour of OS may serve as novel therapeutic targets that can be exploited to improve survival in the metastatic cohort.

Little is known about the factors that drive metastatic progression in OS, however in a number of cancers, intratumoural heterogeneity has been shown to give rise to phenotypic variants within a single tumour that contribute to chemotherapeutic resistance or metastases [3–6]. In this study, we exploited inherent differences in metastatic potential of clonal variants isolated from the same parental cell line to identify potential drivers of OS metastasis. Upregulation of thioredoxin reductase 2 (*TXNRD2*) gene was found exclusively in the highly metastatic clonal variants and its expression was validated against an independent microarray dataset of chemo-naïve OS patient biopsies collected at the time of presentation. The *TXNRD2* gene was upregulated in patients who later progressed to metastatic disease but not in patients whose tumour remained localised.

Thioredoxin reductase (TrxR) belongs to a complex and well regulated system of proteins involved in the reduction and regulation of reactive oxygen species (ROS) in the cytosol and mitochondria [7]. Thioredoxin 1 (*TRX1*) [8], thioredoxin 2 (*TRX2*) [9], *TXNRD1* [10] and *TXNRD2* [11] genes are essential to cell viability since deletions of these genes are embryonic lethal in mice. In mammals, three TrxR proteins are expressed and localised predominantly in the cytoplasm (TrxR1), mitochondria (TrxR2) and testis (TrxR3). One of the major roles of mitochondria is energy metabolism, producing large amounts of ROS and TrxR2, is a key enzyme involved in the regulation of ROS in the mitochondria [12]. Due to its significant role in ROS regulation, the thioredoxin (Trx) system has been an attractive therapeutic target for a number of cancers including pancreatic cancer [13], squamous cell carcinoma (SCC) [14], breast cancer [15, 16] and chronic myeloid leukemia [17–19]. Upregulation of TrxR1 has been associated with lymph node metastasis, and poor prognosis in SCC [14]. In addition, TrxR expression has been shown to promote drug resistance in ovarian cancer cells [20] and radiotherapy resistance in SCC [21].

Auranofin (AF) was the first oral gold(I) compound developed for the treatment of rheumatoid arthritis (RA) [22]. Recent interest in the thioredoxin system as a therapeutic target for cancer has heightened interest in gold compounds, including AF [23]. AF has shown potent cytotoxicity across a panel of 36 cancer cell lines [24]. Often used in combination with existing chemotherapeutic agents [25], AF has shown promising anticancer activity *in vitro*, although little *in vivo* work investigating toxicity has been completed [26]. A central mechanism proposed for the observed anticancer effects of AF [27–29] is the inhibition of TrxR activity through AF's high affinity for seleno groups and therefore the active site of TrxR [30]. AF has been shown to be a specific inhibitor of both cytoplasmic TrxR1 and mitochondrial TrxR2 [30]. The inhibition of TrxR2 can alter the redox balance within a cell, increasing cellular calcium ions, inducing mitochondrial swelling and decreasing mitochondrial membrane permeability. The increased permeability of mitochondrial membranes is accompanied by decreased mitochondrial membrane potential and release of cytochrome c and eventual apoptosis [31, 32]. Therefore in this study, we used AF to target TrxR in OS *in vitro* and *in vivo*. We show that AF inhibits TrxR activity and leads to OS cell death through mitochondrial-mediated apoptosis. More importantly, we show that treatment with AF results in a significant reduction in pulmonary metastasis in an orthotopic mouse model of OS. These data suggest that the use of AF could be a novel therapy to reduce metastasis in OS and thus improve patient survival.

## RESULTS

### Comparative transcriptomic analysis of KHOS clonal variants and patient biopsies identifies novel markers of osteosarcoma metastasis

**Figure 1:**
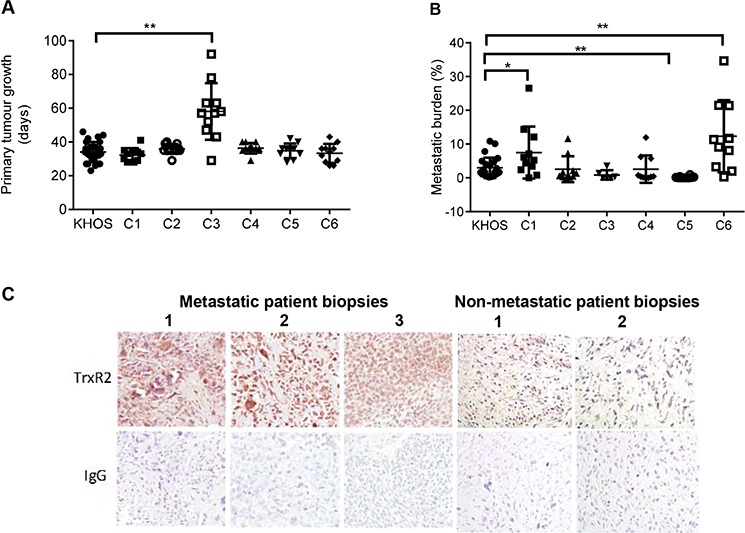
Characterisation of highly and poorly metastatic OS clonal variants and validation of *TXNRD2* **A.** Primary tumour growth of KHOS and clonal variants C1-C6 in an orthotopic mouse model of OS, measured as time in days for the primary tumour to reach 10 mm. **B.** Metastatic burden of KHOS and clonal variants, quantified as percentage area of metastasis/total area of lung in H&E stained lung tissue sections. **C.** Immunostaining of three representative biopsy sections from patients who developed lung metastasis, showing high expression of TrxR2, and three OS patient biopsies who did not develop metastatic disease, showing low expression of TrxR2. Magnification: 20X. Statistical analysis was performed using an unpaired *t*-test. *P < 0.05, **P < 0.01. Bars: SD.

Short term cultures of six clonal variants were generated by randomly plating individual KHOS cells and allowing the clones to expand without selection. Plating efficiency was approximately 50% and hence the generation of clonal variants occurred with negligible selection pressure. BALB/C nude mice were injected intrafemorally with 5 × 10^4^ cells of the clonal variants and tumours allowed to establish. All variants grew with similar kinetics and mice were sacrificed when the size of the primary tumour reached our ethics-approved endpoint of 10 mm. Primary tumours and lungs were removed and metastatic burden quantified. There were significant differences in the metastatic burden between the clonal variants despite the similarities in growth rate and size of the primary lesion (Figure 1A). Metastatic burden was greater than the parental KHOS for two clonal variants (C1 and C6) and lesser for four clonal variants (C2, C3, C4 and C5) (Figure 1B). These data highlight two important points. Firstly, an established human osteosarcoma cell line contains multiple clonal variants that display profoundly different capacity for metastasis. Secondly, the phenotypes of the clonal variants appear to be stable over multiple replicative cycles *in vitro* and *in vivo*.

To identify genes involved in driving metastasis, the transcriptomic profiles of two highly metastatic clones, C1 and C6, and two poorly metastatic clones, C4 and C5, were compared. The set threshold for upregulated genes was greater than 2-fold increase and a B-statistic greater than 3. The analysis identified 31 upregulated genes in the highly metastatic population in at least 2 comparisons. The top 19 upregulated genes are shown in Supplementary Table 1. This list of 19 genes was compared to an independent dataset of OS patient biopsies and non-malignant bone biopsies [33] (Table 1). Using this strategy we were able to show that *TXNRD2* expression was 2.2-fold higher in metastatic clones with a B-statistic of 4.71 (*P* < 0.01). Increased expression of *TXNRD2* (mean intensity normalised to β-actin) was present in approximately 30% of biopsies from patients who developed metastatic disease within 5 years of the initial biopsy. No expression was detected in non-malignant bone and in the biopsies of patients who remained metastasis-free at 5 years. Significantly, our screen also identified vascular endothelial growth factor A (VEGFA) as being increased 13.3-fold (B statistic 4.2) in metastatic clones (Table 1). Expression of *VEGF* was also increased in 40% of metastatic OS patient biopsies, which is consistent with a previous report showing an upregulation of VEGFA in OS patient samples [34]. *TXNRD2* and *VEGF* were two genes found to be highly overexpressed (normalised intensity > 20) exclusively in metastatic but not in non-metastatic or non-malignant bone biopsies.

**Table 1:** Microarray analysis, showing expression of the genes in Supplementary Table 1, of patient biopsies of non-malignant bone (NB), non-metastatic (NM) OS and metastatic (M) OS

Gene	Expression			Percentage (%)		
	NB	NM	M	NB	NM	M
**TXNRD2**	0.00	0.00	29.41	0	0	29
**VEGF**	0.00	3.85	41.78	0	17	42
**ASB1**	0.00	0.00	6.05	0	0	24
**PRSS3**	0.00	6.65	3.74	0	50	35
**LAMB3**	0.00	31.04	105.41	0	50	29
**ITGB2**	1.10	0.00	0.88	20	0	6
**PLAC8**	2.07	0.00	0.21	40	0	6
**S100A4**	9.06	7.22	17.72	80	100	94
**LPXN**	9.23	8.31	22.66	60	83	76
**ORAOV1**	15.69	7.41	9.84	40	83	65
**CDH2**	18.43	7.61	21.37	60	83	65
**STC2**	22.58	9.85	23.74	80	83	65
**TAP1**	36.30	0.87	22.32	60	17	35
**GDF15**	95.78	20.74	38.04	100	100	94

Gene symbols are listed. Expression is the raw probe intensity normalised to β-actin. The percentage of samples expressing the gene is indicated. Genes detected in the clonal variant transcriptomic analysis (Supplementary Table 1), CLIC6, PRRX1, KCNF1 were not detected in any samples and ATP9A and TMEM200A were not present in the Agilent microarray chip used for this analysis and therefore have not been included.

To confirm our transcriptomic analysis data, we performed immunohistochemistry (IHC) on chemo-naïve biopsies of OS patients. Our data confirmed high levels of *TXNRD2* expression in biopsies of patients who later developed metastatic disease (Figure 1C). Furthermore, the identification of *VEGF* as a marker of metastatic potential is supported by the literature and demonstrates the robustness of our screening approach. *VEGF* expression in OS tumours correlates with the development of metastasis and poor prognosis [35]. A clinical trial targeting *VEGF* in OS patients using a humanised mouse monoclonal anti-VEGF antibody, bevacizumab is currently in progress (NCT00667342). These data provide confidence that *TXNRD2* may also be a clinically meaningful target/marker of metastasis in OS.

### Inhibition of thioredoxin reductase by auranofin inhibits OS metastatic phenotype *in vitro* and *in vivo*

**Figure 2:**
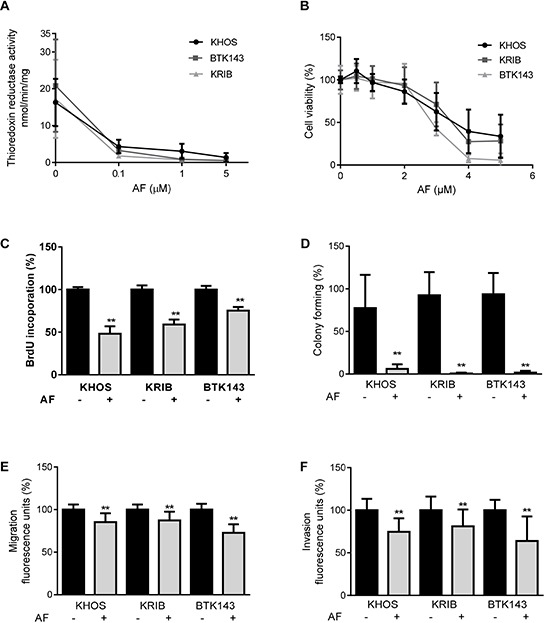
Inhibition of TrxR and the *in vitro* metastatic phenotype in OS by auranofin **A.** Inhibition of TrxR activity in the presence of AF was measured by the reduction rate of (5, 5′-dithiobis (2-nitrobenzoic) acid (DTNB) substrate in an NADPH dependent reaction over 30 min. **B.** AF cytotoxicity was measured by metabolic activity causing cleavage of MTS substrate to formazan, as an indication of cell viability of metastatic osteosarcoma cell lines. **C.** AF decreased proliferation after 24 h treatment, assessed by BrdU incorporation. **D.** Colony forming ability of AF treated cells over 14 days. Cells were stained with Coomassie and quantified by counting colonies with more than 20 cells. **E.** Migration of cells over 24 h through an 8 μM membrane. **F.** Invasion into Matrigel and through an 8 μM membrane, over 48 h. Quantification was performed by measuring Calcein uptake. Statistical analysis was performed using an unpaired *t*-test. *P < 0.05, **P < 0.01. Bars: SD.

Attempts to use shRNA to selectively knockdown *TXNRD2* indicated that *TXNRD1* likely compensated for the knockdown of *TXNRD2* (Supplementary Figure 1). Therefore, we used AF to provide pan-inhibition of both TrxR1 and TrxR2 activity in a suite of metastatic OS cell lines [30]. We chose to examine the effects of AF on OS cell lines because these are composed of mixed populations of cells with different metastatic potentials (i.e. highly metastatic and poorly metastatic subpopulations), which would more closely mimic a heterogeneous OS tumour in the clinic and thus increase the translational significance of our findings. The metastatic osteosarcoma cells showed variable TrxR activity at baseline but treatment with varying doses of AF consistently inhibited TrxR activity in a dose-dependent manner (Figure 2A). Next, we determined whether the dose-dependent inhibition of TrxR activity was due to enzyme inhibition or loss of cell viability (Figure 2B). Low concentrations of AF (0.1 μM and 1 μM) caused 59% and 92% reduction, respectively, of TrxR activity with little evidence of cytotoxicty (Figure 2B). However, at concentrations greater than 2 μM there was a profound and dose-dependent decrease in OS cell viability (Figure 2B). For example, at 5 μM AF reduced cell viability by 95%. Thus, we showed that AF could inhibit TrxR without causing a reduction in cell viability. An AF concentration of 1 μM was chosen to investigate whether inhibition of TrxR activity in metastatic osteosarcoma cells may reduce their metastatic phenotype *in vitro*. Firstly, we measured the effect of TrxR inhibition on metastatic OS cell proliferation by the incorporation of BrdU into DNA. A significant decrease (*P* ≤ 0.0008) in proliferation was observed after treatment with 1 μM AF for 24 h (Figure 2C), whilst the same dose of AF caused a profound and significant (*P* < 0.01) reduction in colony forming ability (Figure 2D). Finally, invasion and migration assays were performed as surrogates for *in vivo* metastatic ability. Metastatic OS cells treated with AF (1 μM) exhibited modest, yet significant (*P* ≤ 0.0007), reductions in migration and invasion (Figure 2E and 2F). Combined, these results indicate that low concentrations of AF inhibit TrxR in metastatic OS cells and induce a profound loss of ability to form colonies. In contrast, higher doses of AF induce a cytotoxic response in metastatic OS cells.

**Figure 3:**
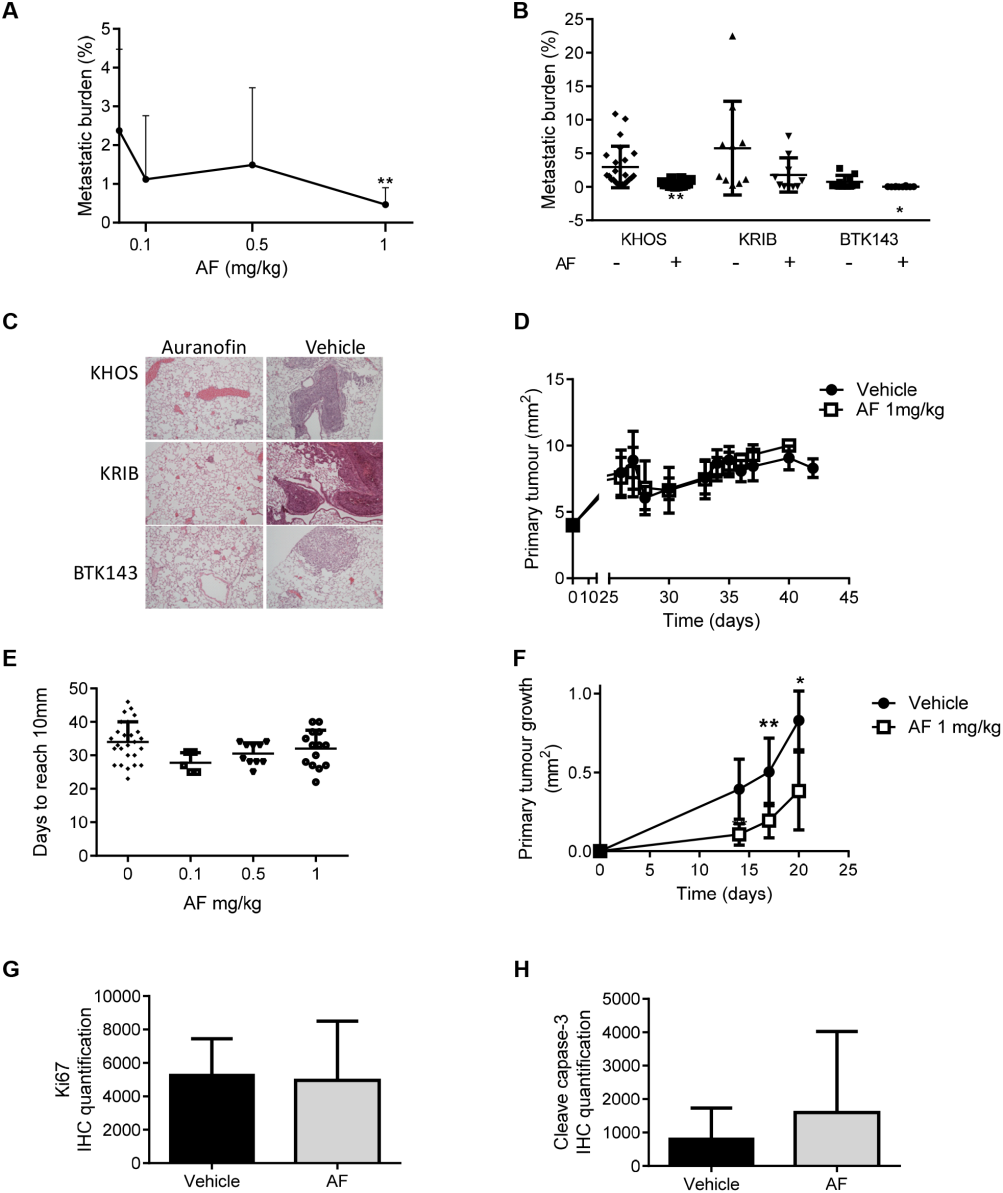
Auranofin is a potent inhibitor of OS metastasis *in vivo* **A.** Dose-dependent reduction of metastatic burden in mice injected orthotopically with KHOS, after treatment with AF. **B.** Reduction of metastatic burden in various metastatic OS cell lines, KHOS, KRIB and BTK143B, injected orthotopically, receiving 1 mg/kg AF, *P = 0.028; **P = 0.004. **C.** Representative H+E stained mouse lung sections of the experiment in **B.** Magnification = 10×. **D.** Orthotopic KHOS primary tumor volume over time in mice treated with 1 mg/kg AF. **E.** Orthotopic primary tumor growth, measured as number of days to reach 10 mm, of mice treated with various AF concentrations. **F.** Subcutaneous primary KHOS tumour growth, measured as tumour volume over time. **G.** Quantification of Ki67 immunostaining of tumours in (F). **H.** Quantification of cleaved caspase-3 immunostaining of subcutaneous tumors treated with vehicle or AF. Staining quantification was performed using Nikon NIS Elements software which uses image segmentation for measurement of colour. Statistical analysis was performed using an unpaired *t*-test. *P < 0.05, **P < 0.01. Bars: SD.

Having shown that AF significantly inhibits the *in vitro* metastatic phenotype of OS, we explored whether AF could inhibit OS pulmonary metastasis *in vivo.* Mice were injected intrafemorally (i.f.) with 5 × 10^4^ KHOS cells and varying doses of AF were administered intra-peritoneally (i.p.), 3 times/week throughout the study. Mice were sacrificed when primary tumours reached 10 mm in diameter. There was a dose-dependent reduction in metastatic burden which reached significance at 1 mg/kg of AF (*P* = 0.0042) compared to vehicle-treated mice (Figure 3A). A similar result was obtained when mice were injected orthotopically with the metastatic cell lines, KRIB or BTK143B, and treated with 1 mg/kg of AF 3 times/week (Figure 3B). Inhibition of metastasis was observed for all cell lines in AF-treated mice, with a significant reduction for KHOS and BTK143B (*P* = 0.0042 and 0.0277, respectively) (Figure 3B). AF-treated mice not only had fewer but also smaller metastatic lesions compared to vehicle-treated mice (Figure 3C). There was no difference in tumour growth or in the time taken by KHOS tumours to reach 10 mm in AF-treated mice at any of the doses tested (Figures 3D and 3E). Our *in vitro* data suggested that AF at low doses inhibited TrxR activity and colony forming efficiency whilst at higher doses induced cytotoxicity (Figure 2). Our *in vivo* data indicated that the doses of AF we used selectively reduced metastatic potential but had little measurable effect on orthotopic primary tumour growth (Figure 3D). This would suggest that the antimetastatic effects we observed with 1 mg/kg AF are below those required for cytotoxicity and are consistent with a TrxR inhibitory dose. However, recent data suggests that the tumour microenvironment can directly suppress cytotoxic responses to known cytotoxic drugs [36]. Thus, the lack of an observed cytotoxic response on the primary OS tumour may not be sufficient to conclude that the OS cells were not exposed to cytotoxic levels of AF. Therefore, we switched the microenvironment in which the OS tumour cells were established and examined the effect of 1 mg/kg AF, i.p. 3 times/week on the growth kinetics of the KHOS cells. Specifically, we established KHOS tumours sub-cutaneously instead of intrafemorally and then treated mice with AF. The sub-cutaneous tumours reached 10 mm in approximately 20 days, whereas the intrafemoral tumours took 30–40 days. However, unlike orthotopic tumours, sub-cutaneous tumours were more sensitive to AF and treated mice exhibited significantly slower primary tumour growth rates (Figure 3F) than vehicle treated mice. Significantly, we noted no impact of AF on the proliferation marker, Ki67 in the AF-treated subcutaneous tumours (Figure 3G). In contrast, we saw a pronounced increase in the apoptotic marker cleaved caspase-3 in the AF treated subcutaneous tumours (Figure 3H). These data indicate two important points. Firstly, 1 mg/kg AF is sufficient to reduce OS tumour growth rates and induce markers of cell death in OS cells *in vivo*. Secondly, given the highly vascular nature of the bone marrow environment one can assume that availability is not limiting. Therefore, our data would suggest that the bone marrow microenvironment may suppress the cytotoxic action of AF on the OS cells. Thus, the selective anti-metastatic action of AF in the orthotopic model of OS may be attributable to the cytotoxic effect of AF on a circulating population of OS cells prior to their establishment of a metastatic focus.

### Auranofin induces ROS-dependent apoptosis in metastatic OS cells

**Figure 4:**
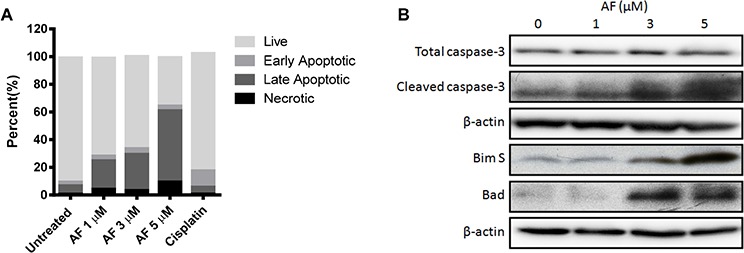
Auranofin treatment induces apoptosis in metastatic OS cells **A.** Apoptotic cell death was determined in KHOS cells treated with AF for 12 h and measured by annexin-V and PI staining using flow cytometry. **B.** Apoptosis marker immunoblot of KHOS cells treated with 0, 1, 3 or 5 μM of AF for 12 h.

Having shown that AF is able to reduce the development of OS metastases in an *in vivo* orthotopic model, and to increase the apoptotic marker, cleaved caspase-3, in AF treated subcutaneous tumours, we sought to elucidate the mechanism for the antimetastatic effects of AF in osteosarcoma. We used flow cytometry to estimate annexin-V and PI staining in OS cells treated with AF. An initial time course experiment was conducted with 1 μM and 5 μM AF (Supplementary Figure 2). Based on this initial experiment we determined that a 12 h exposure was sufficient to induce apoptosis. When KHOS cells were treated with vehicle, 1 μM, 3 μM or 5 μM AF we observed a dose-dependent increase in late apoptotic events as well as a dose-dependent increase in necrotic events after 12 h (Figure 4A). For reference, we have included the apoptotic profiles for OS cells treated with 50 μM cisplatin. Consistent with this we determined that increasing doses of AF induced expression of the cleavage products of caspase-3 as well as increases in the pro-apoptotic markers Bcl-2-like protein 11 (BimS) and Bcl-2-associated death promoter (BAD) (Figure 4B).

**Figure 5:**
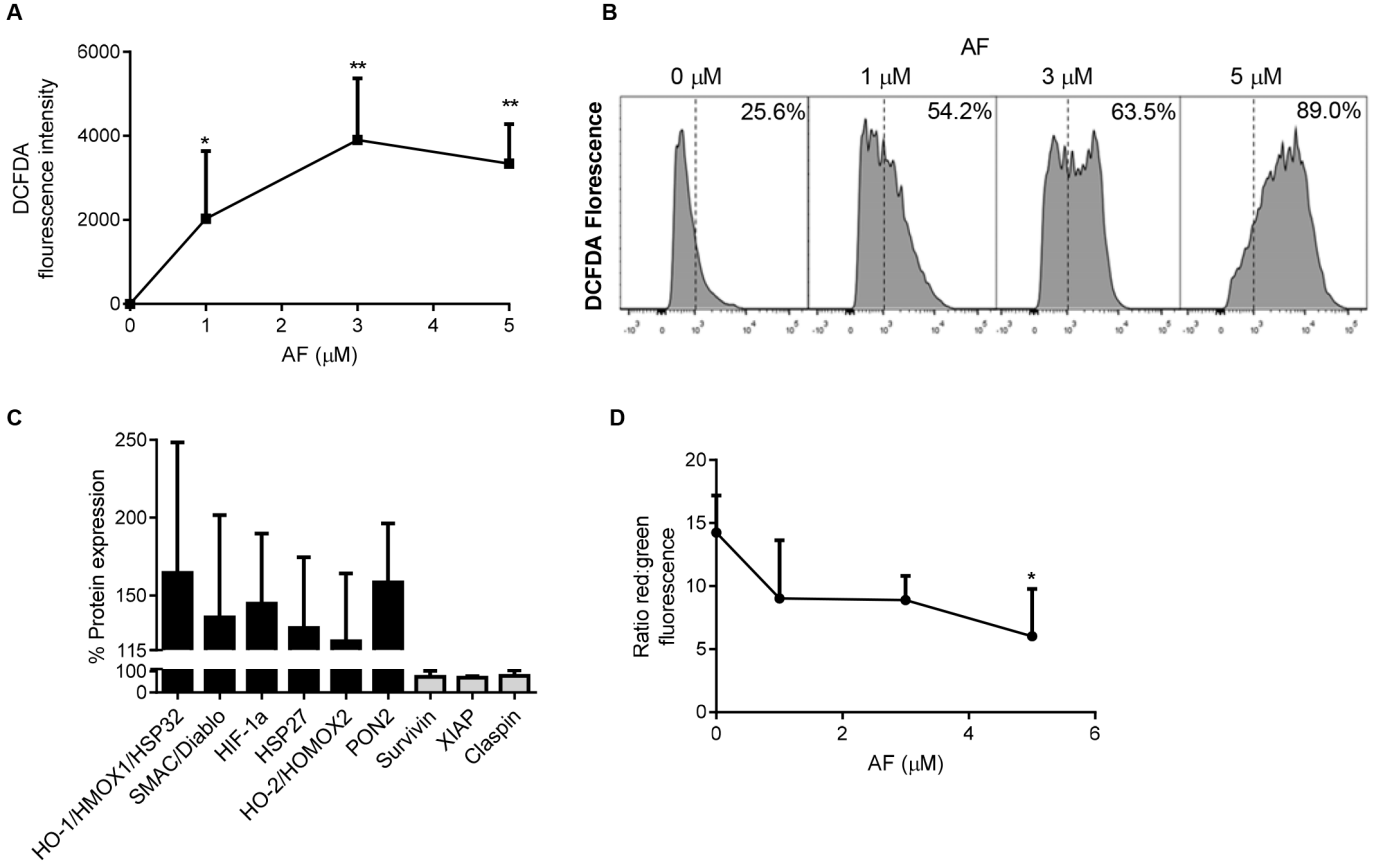
Oxidative stress is increased in OS cells treated with auranofin **A.** Dose-dependent increase in ROS in KHOS cells treated with AF was determined by the oxidation of DCFDA over 15 mins, 37°C. **B.** Flow cytometry determined a shift in the cell population with an increase in the percentage of cells in the DCFDA-high population after 12 h AF treatment. **C.** An apoptosis array of KHOS cells treated with 1 μM AF for 12 h showing upregulation of oxidative stress response proteins and down-regulation of anti-apoptosis proteins. **D.** Increased mitochondrial membrane permeability occurring in cells treated with AF was measured using JC-1 dye incorporation over 15 mins at 37°C. Mitochondrial membrane permeability was visualised using flow cytometry. Functional (energised) mitochondria (red) and dispersed JC-1 dye (green), indicate a loss of mitochondrial membrane potential in AF-treated cells. Statistical analysis was done using an unpaired *t*-test. *P < 0.05, **P < 0.01. Bars: SD.

Apoptosis can occur *via* an extrinsic pathway, mediated by death receptors, and an intrinsic pathway, mediated by the mitochondria [37]. During apoptosis driven by the intrinsic pathway, mitochondria display reduced membrane potential (MMP) referred to as membrane depolarisation (*ΔΨ_m_*), which in turn uncouples oxidative phosphorylation and interferes with the cellular redox potential. Transient mitochondrial depolarisation can be a non-lethal event [31]. However, persisting loss of MMP induces mitochondrial swelling, the release of cytochrome c followed by apoptosis [38]. ROS-driven apoptosis has been linked to AF's anticancer properties [28]. Therefore, we assessed the oxidative burden in OS cells treated with AF for 12 h by measuring the oxidation of the fluorogenic dye, DCFDA, into 2′, 7′ –dichlorofluorescein (DCF) by ROS within the cell [39]. The average intensity of DCFDA/DCF fluorescence in the cell was measured by flow cytometry. We found a dose-dependent increase in oxidative stress in cells treated with AF (Figure 5A). In addition, the percentage of cells exhibiting increased ROS levels also increased with AF treatment (Figure 5B). By interrogation of apoptosis arrays we were able to show that a number of transcription factors associated with an oxidative stress response were upregulated in response to AF, including hypoxia inducible factor 1-alpha (HIF-1α), heme oxygenase-1 (HO-1) and heme oxygenase-2 (HO-2) (Figure 5C). In addition, the pro-apoptotic mitochondrial protein, second mitochondria-derived activator of caspases (SMAC), and the cellular antioxidant paraoxonase 2 (PON2), were also upregulated in response to AF. In contrast, a number of negative regulators of apoptosis were downregulated. These included survivin (BIRC5), x-linked inhibitor of apoptosis protein (XIAP) and claspin (CLSPN) (Figure 5C).

To confirm that AF-induced increases in apoptosis and oxidative burden accompanied the loss of MMP, flow cytometry was used with JC-1 dye. The JC-1 dye accumulates in intact mitochondria and is evident by flow cytometry as red fluorescence at 590 nm. However, loss of MMP is accompanied by loss of JC-1 from the mitochondria and an increase in green fluorescence in the cytoplasm measured at 529nm. Thus, mitochondrial depolarisation is measured by a decrease in the red:green ratio evident by flow cytometry. KHOS cells treated with AF for 12 h showed a dose-dependent decrease in red:green that became significant at 5 μM AF compared to untreated cells (Figure 5D). This increase in MMP was confirmed by showing an increase in red functional (energised) mitochondria in untreated cells while AF treatment (5 μM) increased dispersed JC-1 dye (green), indicating a loss of MMP (Figure 5D). Combined, these data indicate that AF-induced apoptosis of OS cells is ROS-dependent and accompanied by mitochondrial membrane depolarisation.

## DISCUSSION

In this study, we make a number of novel observations: (i) We provide evidence that an established OS cell line contains transcriptomically-different clonal variants that also differ in their ability to metastasise *in vivo*, (ii) OS clonal variants with high metastatic potential and chemo-naïve tumour biopsies from patients who developed metastasis express higher levels of *VEGFA* and *TXNRD2* than non-metastatic OS, (iii) inhibition of TrxR2 with a clinically available inhibitor, the gold(I) complex, AF, reduces the development of pulmonary metastasis, (iv) we provide evidence which suggests the bone marrow microenvironment may protect primary OS lesions from the cytotoxic action of AF *in vivo* and (v) AF causes the activation of an ROS-dependent intrinsic apoptotic pathway in OS cells.

Osteosarcoma is known to be a heterogeneous disease with clinical evidence for inter-tumoural heterogeneity (e.g. different histopathological subtypes) and intra-tumoural heterogeneity. Whilst there are no published studies on the existence of genetically distinct clonal variants within a single OS lesion, the presence of intra-tumoural heterogeneity within OS is inferred by the varied responses of patient lesions to chemotherapy. In contrast, there is definitive proof of genetically distinct clonal variants within many common cancer types [6, 40, 41] which have been shown to drive phenotypic variability with respect to drug resistance or tumour initiating activity. In the present study, we demonstrate that clonal variants exist within an established human OS cell line and differ in their transcriptomes as well as in their ability to metastasise *in vivo*. Significantly, we show that highly metastatic variants express high levels of *TXNRD2* and *VEGFA* which were also highly expressed in a subset of chemo-naive OS biopsies. These data suggest that the metastatic potential of a single lesion may be determined by a subset of cells that possess highly metastatic behaviour. Given that not all clonal variants were highly metastatic, yet all retained the ability to initiate a tumour and grow, we would speculate that the metastatic phenotype may emerge at a later point in tumour evolution than does the ability to initiate a tumour. If true, this would indicate that drugs able to inhibit the metastatic phenotype (e.g. AF) should be deployed as early as possible following diagnosis. Phenotypic heterogeneity can arise from genetic or epigenetic differences between variants or may simply reflect differences in response to microenvironmental cues or inherent phenotypic plasticity (e.g. an EMT phenotype) [5]. Whilst we have not performed exome or whole genome sequencing on our clonal variants, it is clear that the phenotype is stable enough to be preserved through multiple *in vitro* and *in vivo* replication cycles, which is consistent with a genetic/epigenetic basis for the difference.

A significant finding in our study was the observation that AF is able to reduce the development of OS metastases *in vivo*. We propose that the direct cytotoxic/cytostatic activity of AF is the major contributor to its antimetastatic activity. Supporting this we show that AF causes a dose-dependent and ROS-dependent increase in apoptosis. We also demonstrated that non-cytotoxic doses of AF were able to inhibit TrxR but had only modest (yet significant) effects on *in vitro* migration or invasion, but showed profound effects on colony forming efficiency of OS cells. Finally, we show that AF treatment of mice bearing subcutaneous OS lesions resulted in reduced tumour growth and evidence of increased intratumoural cleavage of caspase-3. Thus, we have *in vitro* and *in vivo* data indicating that AF is able to induce apoptosis and reduce colony formation at doses which produce only modest changes in OS cell migration and invasion. This would suggest that AF's antimetastatic effect is not due to the inhibition of migration or invasion.

Another significant finding of our study was that OS cells injected intrafemorally were able to establish and grow in the presence of a known cytotoxic dose of AF but displayed profoundly reduced metastatic potential. These data suggest that the bone marrow microenvironment may protect OS cells from the cytotoxic effects of AF whilst OS cells that exit the bone marrow may be vulnerable to the cytotoxic action of AF. In support of this, we have previously shown that osteoclasts provide an environment which prevents metastasis and suppresses OS cell migration [42, 43]. Similarly, it has also been shown that the microenvironment within the bone and lymph nodes is able to promote the survival of chronic lymphocytic leukaemia cells [44]. Moreover, recent studies in melanoma (HGF secreted by stromal cells inhibits cytotoxicity due to vemurafenib) [45] have shown that tumour stroma is a major determinant of sensitivity of primary cancer cells to cytotoxic agents. Thus, the bone marrow microenvironment appears to actively inhibit the sensitivity of OS cells to AF-induced cytotoxicity, while the metastatic or circulating tumour cell population remains sensitive.

Thus far, TrxR has proven to be a successful drug target, with a number of gold compounds, particularly AF, displaying potent anticancer activity in models of lung cancer [46], ovarian cancer [20] and breast cancer [16]. However, its role in OS had never been investigated. TrxR2 is a mitochondrial selenoenzyme that has been shown to be involved in the regulation of metabolic and oxidative stress in the cell [12]. In addition, increased expression of TrxR2 has been shown to support tumour growth and protect tumour cells from fluctuating levels of ROS and oxidative stress [47–50]. The mitochondria play a fundamental role in energy metabolism as well as regulation of cellular redox levels, ion homeostasis and mitochondrial mediated apoptosis [38]. ROS can initiate signalling pathways involved in the regulation of cell survival and cell death. High levels of ROS can cause DNA damage and protein oxidation leading to necrosis and apoptosis. TrxR2 also plays a protective role through anti-apoptotic activity in ROS- stressed cells as well as impacting chemotherapeutic resistance [50]. In the present study we show that total inhibition of TrxR activity in OS cells by AF is accompanied by increases in ROS and the activation of the intrinsic apoptosis pathway. AF is a well known TrxR inhibitor, with a higher level of cytotoxicity in cancer cells compared to other gold compounds such as aurothioglucose. This has been linked to AF's greater lipid solubility, allowing better absorption into both the cytoplasm and mitochondria [51]. Thus, AF-induced oxidation of thioredoxin causes a redox imbalance and resultant accumulation of ROS and subsequent activation of the intrinsic apoptotic pathway resulting in mitochondrial dysfunction and cell death. In this regard, we showed that AF-induced apoptosis was accompanied by increased cellular levels of ROS, followed by the upregulation of master regulators of the oxidative stress response such as the transcription factor HIF1-α and other mediators of oxidative stress response including HO-1 and SMAC. Similarly, these responses to oxidative stress triggered a cascade of events resulting in increases in both BimS and Bad protein expression followed by mitochondrial mediated apoptosis in KHOS cells.

Finally, we show that AF induces OS cell apoptosis *in vitro* and *in vivo*. Our data indicates that inhibition of TrxR activity is central to the apoptotic effects of AF but that high concentrations of AF may directly contribute to oxidative stress and hence further enhance apoptotic responses. This is supported by our data showing that OS cells are tolerant of relatively large amounts of TrxR2 inhibition after which the capacity of the cellular pool of TRX is unable to cope with the increasing oxidative stress. Hence, OS cells remain viable at lower doses of AF. Significantly, doses of AF above those required to maximally inhibit TrxR continue to increase the apoptotic indices of OS cells which is accompanied by increased oxidative stress. These data suggest that AF anticancer activity required the inhibition of TrxR activity to a level which also results in oxidative damage. This is consistent with the findings of Du *et al*. who showed that oxidation of TRX1 substrate was correlated with cell death rather than with TrxR1 and TrxR2 activity inhibition [51], suggesting that disruption of cellular redox balance is essential to increase ROS and cause cell death.

## MATERIALS AND METHODS

### Cell culture

Human OS cell lines KHOS, KRIB and BTK143 were maintained in Dulbecco's Modified Eagle Medium (DMEM) supplemented with 10% fetal bovine serum (FBS), 100 units/ml penicillin-streptomycin and 2.92 mg/ml L-glutamine, and cultured at 37°C in a humidified incubator with 5% CO_2_. These conditions were used in all assays unless otherwise stipulated.

### Thioredoxin reductase activity assay

TrxR activity was measured using a TrxR activity kit (Abcam), following the manufacturers' instructions. Cells were treated with auranofin (Sigma-Aldrich) at the stated concentrations for 24 h, collected and lysed using dounce homogenization for use in the TrxR kit.

### MTS assay

Cells were seeded in a 96-well plate at a concentration of 5000 cells/100 μL/well and incubated for 24 h. Medium was replaced with medium containing AF at the stated concentrations. Cells were incubated for 48 h. Cell viability was determined by addition of 20 μL of Cell Titer 96 Aqueous One Solution (Promega), incubation for 2–4 h, and measuring absorbance at 490 nm.

### Proliferation assay

Cells were seeded in a 96-well plate at 5000 cells/100 μL/well and treated with AF at the stated concentrations for 12 h. Proliferation was determined by a colorimetric ELISA of incorporation of the thymidine analogue, 5-bromo-2′deoxyuridine (BrdU) into cells during DNA synthesis (Roche Diagnostics), following the manufacturer's instructions.

### Migration and invasion assays

The metastatic OS cell lines were seeded in serum-free medium, at 2 × 10^5^ cells/mL, in 100 μL, into the insert with a permeable support 8 μm polycarbonate membrane, of a 24-well transwell plate (Corning). AF was added to the cells at the stated concentrations. Migration was induced by addition of medium containing 20% FBS to the bottom chamber. Migration was quantified after 24 h. Invasion assays were performed similarly but with the addition of 50 μL of Matrigel (BD Biosciences) diluted 1:6 with medium to the transwell insert, and with an incubation time of 48 h. Cells that had migrated to the underside of the membrane were labeled by incubation with 8 μM Calcein AM (Sigma-Aldrich) in serum-free medium for 45 min at 37°C. Cells were dislodged from the membrane by incubation in 0.5% trypsin/EDTA for 10 min at 37°C with gentle intermittent shaking. A 200 μL sample of cell suspension was thoroughly mixed and taken for fluorescence quntification at 485 nm excitation, 520 nm emission (FLUOstar OPTIMA, BMG Labtech).

### Colony forming assay

Colony forming assays were performed as described [52]. Briefly, known numbers of cells were seeded at varying densities in a 6-well plate. Once cells were attached, 1 μM AF was added and cells were allowed to grow for 10 days. Cells were fixed and stained with Coomassie Blue. Colony forming efficiency was expressed as a percentage of the number of colonies counted on untreated control plates.

### Immunohistochemistry (IHC)

Formalin-fixed, paraffin-embedded sections of mouse OS tumours and lungs were incubated with antibody to TrxR2 (Sigma-Aldrich) or Rabbit IgG (Dako), at 0.9 μg/mL, overnight at 4°C. Detection and visualization was performed with Starr Trek Universal HRP Detection System and Cardassian DAB Chromogen (Biocare Medical), following the protocols recommended by the manufacturer.

### Measurement of apoptosis

OS cells were seeded in 6-well plates at 1 × 10^5^ cells/mL (2.5 mL/well) and incubated overnight. Cells were treated with AF at the stated concentrations for 12 h. FITC Annexin V Apoptosis Detection Kit with PI (Biolegend) was used to measure cell death, as per the manufacturer's instructions. Cells were analysed using flow cytometery on a FACS Canto acquisition instrument (Beckman Coulter). Data were analysed with Flow Jo Software (TreeStar).

### Apoptosis array

Cells were seeded in 10 cm dishes at 2.5 × 10^6^ cells/mL and incubated overnight. Cells were treated with 1 μM AF for 12 h and lysates were collected. Lysates were assayed on a Human Apoptosis Antibody Array Kit (R&D Systems) according to the manufacturer's protocol. Spots were quantified using a gel imager (Vilber Lourmat) and Vilber Bio-1D analysis software (Fisher Biotec).

### Isolation of OS clonal variants

KHOS cells, previously shown to be metastatic *in vivo* [43] were seeded in 24-well plates at a density of less than 1 cell/well. Individual cells were allowed to divide and grow into colonies over several days. Cells were passaged into larger plates and eventually tissue culture flasks, before freezing in liquid nitrogen. Six clonal variants (labeled C1-C6) were selected at random and individually injected intrafemorally into female 6-week-old BALB/c nude mice as described in the section **In vivo* metastasis studies*.

### Gene expression analysis

Highly metastatic (C1, C6) and poorly metastatic (C4, C5) KHOS clonal variants were analysed in triplicate on a Human HT-12 v4 Expression BeadChip (Illumina) after amplification using the Illumina^®^ TotalPrep RNA Amplification Kit (Life Technologies). Raw gene expression values were extracted with BeadStudio data analysis software (Illumina). Data quality was determined using the positive and negative control probes as well as by inspection of the distributions of probe intensities. Data were normalised using the quantile normalisation method in the Lumi package of Bioconductor. Stringent analysis using a B-statistic > 3 was used to select genes that had ≥ 95% chance of being differentially expressed between the metastatic and non-metastatic groups. The transcriptomic analysis of chemo-naïve patient biopsies has been described previously [43].

### *In vivo* metastasis studies

All animal experimentation was approved by, and carried out in strict accordance with the recommendations of The University of Queensland Health Sciences Ethics Committee (Approval Number: UQDI/PAH/292/12/NHMRC). Five to fourteen female 6–8 week-old BALB/c nude mice were used in each group and each experiment was repeated independently at least once. Cells were injected intra-femorally (i.f.) at 50,000 cells/10 μL/injection. Sub-cutaneous injections were performed with 1 × 10^5^ cells/100 μL. In studies involving administration of AF (1 mg/kg) mice were injected intraperitoneally (i.p), 3 times/week, beginning day 0 after i.f. injection. Mice were euthanised when tumours reached 10 mm in diameter. Lungs were perfused with 4% paraformaldehyde and H&E stained. Metastatic burden was quantified as the total area of metastasis divided by the total area of lung, using Nikon NIS-Elements software (Nikon).

### Western blotting

Cells were treated with AF at various concentrations for 12 h and protein extracts perepared. Samples were resolved using 10% SDS-PAGE. Proteins were detected with antibodies to TXNRD1 1:1000 (Abcam); TXNRD2 1:1000; β-actin 1:6,000 (all from Sigma-Aldrich); BIM 1:1000; BAD 1:1000; Cleaved caspase-3 1:1000; Total caspase-3 1:1000 (all from Cell Signaling). Horseradish peroxidase conjugated secondary goat anti-rabbit (Santa Cruz) and rabbit anti-mouse (BD Pharmingen) were used for detection.

### DCFDA assay

Fluorogenic dye 2′,7′ –dichlorofluorescin diacetate (DCFDA) (Sigma-Aldrich) at 25 mg/mL was used as an indicator ROS activity [39]. Using DCFDA, hydroxyl, peroxyl and other ROS production was measured in cells treated with AF. Cells were seeded in 6-well plates at 1 × 10^5^ cells/mL (2.5 mL/well) and allowed to adhere for 4 h. Cells were treated with AF at the stated concentration and incubated for 12 h at 37°C. Cells were collected and washed in PBS. DCFDA was added at 1:2000 to a final concentration of 12.5 μM, and incubated for 30 min at 37°C. Florescence intensity was measured by flow cytometry with excitation 495 nm and emission 529 nm (BD Biosciences).

### Assessment of mitochondrial membrane potential

JC-1 dye (AdipoGen) was used as an indicator of mitochondrial membrane permeability. The ratio of green to red fluorescence was used as a measure of mitochondrial membrane potential. Cells were seeded in 6-well plates at 1 × 10^5^ cells/mL (2.5 mL/well) and allowed to adhere for 4 h. Cells were treated with AF at the stated concentration and incubated for 12 h at 37°C. Cells were collected and washed in PBS. Cells were stained with 2 μM JC-1 for 15 min at 37°C. The AF treated cells were stained with 2 μM JC-1 for 15 min at 37°C and analysed by flow cytometry using 488 nm excitation and 530nm/585 nm emission bandpass filters (BD Biosciences).

### Statistical analysis

All statistical tests were performed using GraphPad Prism using column analysis and a parametric unpaired two-tailed *t*-test analysis.

## SUPPLEMENTARY FIGURES AND TABLE


