# Supplementary material for: Auranofin is a potent suppressor of osteosarcoma metastasis
Source: Oncotarget. 2015 Nov 9;7(1):831–44. doi: 10.18632/oncotarget.5704 (PMC4808036; doi:10.18632/oncotarget.5704)
Supplement: Supplementary file 1 [file oncotarget-07-0831-s001.pdf]

SUPPLEMENTARY FIGURES AND TABLE

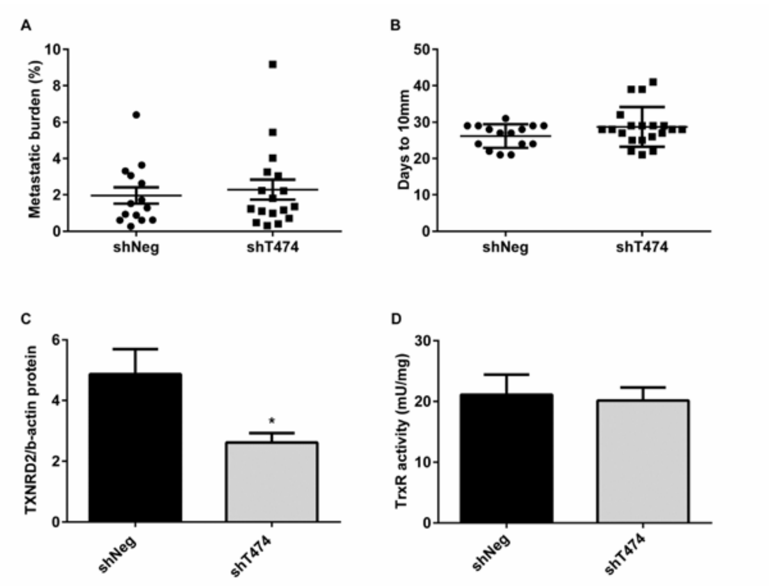

Supplementary Figure S1: TXNRD1 compensates for activity in TXNRD2 knockdown. A. Metastatic burden and **B.** primary tumour growth of TrxR2-silenced cells (shT474) is unchanged in an orthotopic mouse model of OS. **C.** Immunoblot quantification of TrxR2 protein knockdown in the tumours in (A) and (B), corrected for B-actin expression. $^*P < 0.05$. **D.** Total thioredoxin activity in the TrxR2 knockdown cells is unchanged.

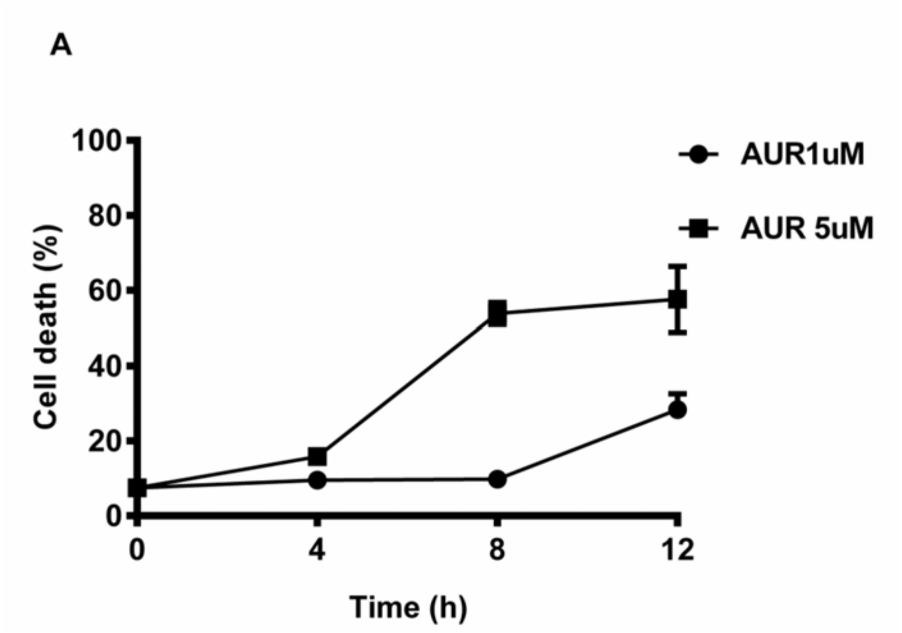

Supplementary Figure S2: Time course of auranofin-induced apoptosis in OS cells. KHOS cells were treated with 1 and 5 µM AF over 12 hours to determine the highest level of cell death as measured by Annexin-V and PI staining.

Supplementary Table S1: Top 19 genes overexpressed in a comparative transcriptomic analysis of highly and poorly metastatic clonal variants of OS

Gene	Symbol	B statistic	FC
**Stanniocalcin 2**	STC2	12.53	6.81
**Cadherin 2, type 1, N-cadherin (neuronal)**	CDH2	15.63	6.45
**Paired related homeobox 1**	PRRX1	8.13	5.07
**Vascular endothelial growth factor A**	VEGFA	13.26	4.17
**S100 calcium binding protein A4**	S100A4	8.93	4.12
**Growth differentiation factor 15**	GDF15	8.18	3.77
**Oral cancer overexpressed 1**	ORAOV1	8.06	2.99
**Integrin, beta 2**	ITGB2	9.29	2.97
**Protease, serine, 3**	PRSS3	6.16	2.90
**Potassium voltage-gated channel, subfamily F, member 1**	KCNF1	6.86	2.84
**Ankyrin repeat and SOCS box containing 1**	ASB1	10.37	2.73
**Leupaxin**	LPXN	6.96	2.65
**ATPase, class II, type 9A**	ATP9A	9.03	2.59
**Placenta-specific 8**	PLAC8	5.43	2.55
**Transporter 1, ATP-binding cassette, sub-family B (MDR/TAP)**	TAP1	8.24	2.53
**Laminin, beta 3**	LAMB3	5.38	2.52
**Transmembrane protein 200A**	TMEM200A	7.87	2.34
**Chloride intracellular channel 6**	CLIC6	7.62	2.28
**Thioredoxin reductase 2**	TXNRD2	4.71	2.19

Illumina gene names and symbols are listed. A B-statistic > 3 indicates a 95% chance that the gene is truly differentially expressed. FC = fold change.
